# Comparative efficacy and safety of atezolizumab and bevacizumab between hepatocellular carcinoma patients with viral and non‐viral infection: A Japanese multicenter observational study

**DOI:** 10.1002/cam4.5337

**Published:** 2022-10-13

**Authors:** Takeshi Hatanaka, Satoru Kakizaki, Atsushi Hiraoka, Toshifumi Tada, Masashi Hirooka, Kazuya Kariyama, Joji Tani, Masanori Atsukawa, Koichi Takaguchi, Ei Itobayashi, Shinya Fukunishi, Kunihiko Tsuji, Toru Ishikawa, Kazuto Tajiri, Hironori Ochi, Satoshi Yasuda, Hidenori Toyoda, Chikara Ogawa, Takashi Nishimura, Noritomo Shimada, Kazuhito Kawata, Hisashi Kosaka, Takaaki Tanaka, Hideko Ohama, Kazuhiro Nouso, Asahiro Morishita, Akemi Tsutsui, Takuya Nagano, Norio Itokawa, Tomomi Okubo, Taeang Arai, Michitaka Imai, Atsushi Naganuma, Yohei Koizumi, Shinichiro Nakamura, Kouji Joko, Masaki Kaibori, Hiroko Iijima, Yoichi Hiasa, Takashi Kumada, the Real‐life Practice Experts for HCC (RELPEC) Study Group, and HCC 48 Group (hepatocellular carcinoma experts from 48 clinics in Japan)

**Affiliations:** ^1^ Department of Gastroenterology Gunma Saiseikai Maebashi Hospital Maebashi Japan; ^2^ Department of Clinical Research National Hospital Organization Takasaki General Medical Center Takasaki Japan; ^3^ Department of Gastroenterology and Hepatology Gunma University Graduate School of Medicine Maebashi Japan; ^4^ Gastroenterology Center Ehime Prefectural Central Hospital Matsuyama Japan; ^5^ Department of Internal Medicine Japanese Red Cross Himeji Hospital Himeji Japan; ^6^ Department of Gastroenterology and Metabology Ehime University Graduate School of Medicine Ehime Japan; ^7^ Department of Gastroenterology Okayama City Hospital Okayama Japan; ^8^ Department of Gastroenterology and Hepatology Kagawa University Kagawa Japan; ^9^ Division of Gastroenterology and Hepatology, Department of Internal Medicine Nippon Medical School Tokyo Japan; ^10^ Department of Hepatology Kagawa Prefectural Central Hospital Takamatsu Japan; ^11^ Department of Gastroenterology Asahi General Hospital Asahi Japan; ^12^ Premier Departmental Research of Medicine Osaka Medical and Pharmaceutical University Osaka Japan; ^13^ Center of Gastroenterology Teine Keijinkai Hospital Sapporo Japan; ^14^ Department of Gastroenterology Saiseikai Niigata Hospital Niigata Japan; ^15^ Department of Gastroenterology Toyama University Hospital Toyama Japan; ^16^ Center for Liver‐Biliary‐Pancreatic Disease Matsuyama Red Cross Hospital Matsuyama Japan; ^17^ Department of Gastroenterology and Hepatology Ogaki Municipal Hospital Ogaki Japan; ^18^ Department of Gastroenterology Japanese Red Cross Takamatsu Hospital Takamatsu Japan; ^19^ Department of Internal Medicine, Division of Gastroenterology and Hepatology Hyogo College of Medicine Nishinomiya Japan; ^20^ Division of Gastroenterology and Hepatology Otakanomori Hospital Kashiwa Japan; ^21^ Hepatology Division, Department of Internal Medicine II Hamamatsu University School of Medicine Hamamatsu Japan; ^22^ Department of Surgery Kansai Medical University Hirakata Japan; ^23^ Department of Gastroenterology National Hospital Organization Takasaki General Medical Center Takasaki Japan; ^24^ Department of Nursing Gifu Kyoritsu University Ogaki Japan

**Keywords:** etiology, immune checkpoint inhibitor, non‐alcoholic steatohepatitis, propensity score matching, real‐world data

## Abstract

**Aim:**

This study compared the efficacy and safety of atezolizumab and bevacizumab (Atez/Bev) in patients with viral and non‐viral infection in clinical settings.

**Methods:**

We conducted the retrospective cohort study of 323 BCLC stage B or C hepatocellular carcinoma (HCC) patients with Child‐Pugh class A, and a performance status of 0 or 1 who started Atez/Bev from September 2020 to December 2021 at 22 institutions in Japan. Patients with viral infection was defined as those who were either serum anti‐HCV‐ Ab or HBs‐Ag‐positive, while patients with non‐viral infection was defined as those who were both serum anti‐HCV Ab‐ and HBs‐Ag‐negative. We constructed a propensity‐score‐matched cohort to minimize the risk of observable potential confounders.

**Results:**

Propensity score matching produced 126 matched pairs for patients with viral versus non‐viral infection. After matching, the significant differences in baseline demographic features did not exist between the two groups. The objective response rate was 20.6% and 24.6% in viral‐ and non‐viral‐related HCC patients, respectively, without a significant difference (*p* = 0.55). The disease control rate was not also significantly different (68.3% vs 69.0%, *p* = 1.00). The median progression‐free survival was 7.0 months (95% confidence interval [CI] 6.0–9.6) and 6.2 months (95% CI 5.1–7.8) in patients with viral and non‐viral infection, and the 12‐month survival rates were 65.5% (95% CI 50.8–76.8) and 71.7% (95% CI 57.3–81.9) in those with viral and non‐viral infection, respectively, which were not significantly different (*p* = 0.33, *p* = 0.38). No significant difference in treatment‐related adverse events was found between the two groups.

**Conclusions:**

Our etiology‐based study demonstrated that Atez/Bev showed good efficacy and safety for HCC patient with non‐viral infection as well as those with viral infection.

## Graphical abstract

The survival curve of HCC patients with viral and non‐viral infection treated with atezolizumab and bevacizumab. No significant differences were found between the two groups (p = 0.38, hazard ratio 0.76, 95% CI 0.42–1.39).

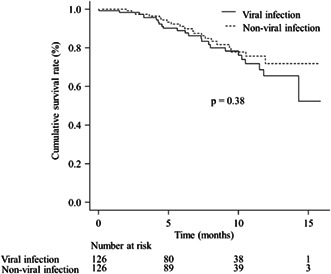

## 1 INTRODUCTION

According to a pivotal randomized control trial (RCT),^1^ atezolizumab combined with bevacizumab (Atez/Bev), which is combination therapy with a monoclonal antibody targeting programmed death‐ligand 1 and monoclonal antibody against vascular endothelial growth factor (VEGF) A, significantly improved the overall survival (OS) and progression‐free survival (PFS) in comparison to sorafenib. Atez/Bev is currently recommended as the first‐line treatment for patients with advanced hepatocellular carcinoma (HCC) under recent guidelines.^2^, ^3^

In a subgroup analysis of the above RCT, the hazard ratio (HR) for death in patients receiving Atez/Bev was 0.91 (95% confidence interval [CI] 0.52–1.60), 0.51 (95% CI 0.32–0.81), and 0.43 (95% CI 0.22–0.87) for non‐viral‐, hepatitis B virus (HBV)‐, and hepatitis C virus (HCV)‐related HCC patients, respectively.^1^ Based on the updated report concerning the efficacy and safety of this RCT, the median OS was 17.0 months (95% CI 11.7–22.8) and 18.1 months (95% CI 11.7–26.3) in patients with non‐viral infection treated with Atez/Bev and sorafenib, respectively, resulting in an HR of 1.05 (95% CI 0.68–1.63) for death with Atez/Bev.^4^

Two meta‐analyses of immune checkpoint inhibitor (ICI) trials showed that the pooled HR for the OS in viral‐related HCC patients receiving an ICI was lower than in those receiving standard‐care treatment.^5^, ^6^ These two studies indicated that patients with viral infection benefitted from ICI treatment, while those with non‐viral infection did not.^5^, ^6^ Our previous study^7^ reported the initial experience of Atez/Bev treatment, showing that the initial therapeutic response (radiologically evaluated at 6 weeks after the starting Atez/Bev) was not significantly differed between the patients with viral and non‐viral infection (10.8% vs 10.4%, *p* = 0.96, *n* = 171). However, we did not evaluate the PFS, OS and adverse events (AEs) owing to the short observation period. In addition, our previous report^7^ did not correct imbalances in baseline characteristics between viral‐, and non‐viral‐related‐HCC patients. Accordingly, whether or not underlying liver etiologies can predict the therapeutic response to Atez/Bev remains unclear.

In the current study, we expanded our cohort with long observation period, and compared the therapeutic outcome of Atez/Bev in patients with viral and non‐viral infection in clinical settings using propensity score matching.

## 2 METHODS

### 2.1 Patient population

**FIGURE 1:**
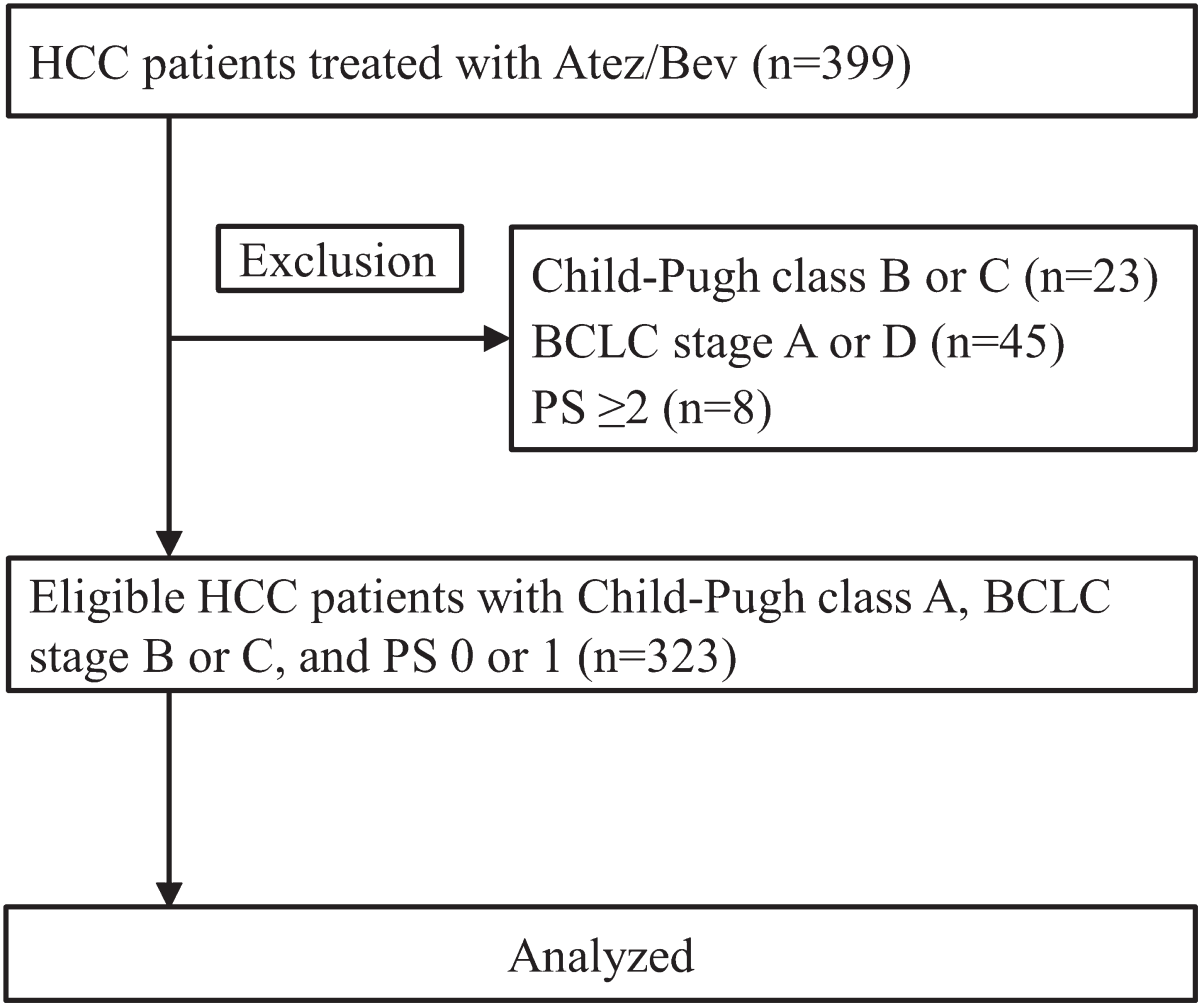
Flow diagram of the eligible hepatocellular carcinoma patients

In the present retrospective cohort study, 399 HCC patients who received Atez/Bev from September 2020 to December 2021 were included. The study enrolled patients at 22 different institutions in Japan. We excluded patients with a poor liver function (that is, Child‐Pugh class B or C; *n* = 23), those with Barcelona Clinic Liver Cancer (BCLC) stage A or D (*n* = 45), and those with a performance status (PS) ≥2 (*n* = 8). Accordingly, we included the remaining 323 HCC patients with Child‐Pugh class A, BCLC stage B or C, and PS ≤1 in the present study (Figure 1). HCC was diagnosed based on histological data obtained during the clinical course or the typical findings on radiological imaging including enhanced dynamic computed tomography (CT) and dynamic magnetic resonance imaging (MRI).^8^

### 2.2 Definitions of viral and non‐viral infection

When participated patients tested positive for anti‐HCV antibody (anti‐HCV Ab), cause of HCC was attributed to chronic HCV infection. When patients tested positive for hepatitis B virus surface antigen (HBs‐Ag), HCC was attributed to chronic HBV infection. Patients with viral infection were defined as those with either anti‐HCV Ab or HBs‐Ag seropositivity, while patients with non‐viral infection were defined as those with both serum anti‐HCV Ab and HBs‐Ag seronegativity. If patients with non‐viral infection had a presence and/or history of habitual alcohol consumption (≥60 g/day), HCC was attributed to chronic alcoholic liver injury. The diagnosis of non‐alcoholic fatty liver disease (NAFLD) was performed based on the pathological examination or clinical findings; that is, findings of fatty liver, medical interview of low habitual alcohol intake (<30 g/day in males and <20 g/day in female), and metabolic comorbidities.

### 2.3 Treatment procedure

Before Atez/Bev treatment, we evaluated the preserved liver function, tumor extension, and PS to determine the BCLC stage.^2^ In addition to the Child‐Pugh classification, we also used the albumin‐bilirubin (ALBI) score^9^ and modified ALBI (mALBI) grade^10^ to assess the liver function. Tumor extension was assessed based on findings of radiological imaging including dynamic CT or MRI.

Patients intravenously received 1200 mg of atezolizumab followed by 15 mg/kg of bevacizumab on the same days every 3 weeks. Atez/Bev was continued until disease progression was observed or unacceptable AEs developed. The tumor response after initiation of Atez/Bev was evaluated by Response Evaluation Criteria in Solid Tumors version 1.1 (RECIST v.1.1). We evaluated the degree of AEs according to The Common Terminology Criteria for Adverse Events version 5.0. When treatment‐related AEs were observed, we interrupted or discontinued each drug according to the guidelines for Atez/Bev treatment provided by the manufacturer.

### 2.4 Statistical analyses

Categorial variables were presented as the counts (percentage) and numerical variables as median [interquartile range]. The differences between categorial variables were calculated using the χ^2^ test, and Fisher's exact, if appropriate. Numerical variables were compared using Mann–Whitney *U*‐test. Given the differences in the patient characteristics associated with efficacy and safety between the eligible patients with viral and non‐viral infection, a propensity‐score‐matched cohort was identified to minimize the risk of observable potential confounder. The propensity score for each patient was estimated by generating a logistic regression model. The matching variables included the age, gender, BCLC stage, and mALBI grade. We used 1:1 nearest neighbor matching without replacement and adopted caliper width of 0.2 of standard deviations.

The PFS was measured between the starting date of Atez/Bev and the date of disease progression or death, whichever came first. The OS was calculated from the initiation date of Atez/Bev to death or to the date of last follow‐up. The survival outcome was assessed using the Kaplan–Meier method and the log‐rank test was used to compare the differences between subgroups. The up‐to‐seven criteria was defined as the sum of largest tumor size (cm) and number of tumors in BCLC intermediate stage HCC patients. All of the reported *p*‐values were two‐sided. If it was less than 0.05, a result was considered significant. All statistical analyses were conducted using EZR V. 1.53 (Saitama Medical Center, Jichi Medical University, Saitama, Japan).^11^

## 3 RESULTS

### 3.1 Patient characteristics before and after propensity matching

Table 1 shows the baseline features of all eligible patients before propensity matching. The median age of the entire cohort was 73 (68.0–79.0) years old, with about 80% of patients being male. 103 (31.9%) and 62 (19.2%) patients were HCV Ab, and HBs‐Ag positive, respectively. Of the remaining non‐B, non‐C, patients, 59 (18.3%), 56 (17.3%), and 43 (13.3%) patients were determined to alcohol, NAFLD, and others, respectively. Therefore, 165 (51.1%) and 158 (48.9%) patients were allocated to the viral and non‐viral infection groups, respectively. With respect to antiviral therapy, 77 HCV patients (76.6%) achieved sustained virological response and 40 HBV patients (75.5%) have already received nucleoside analogue therapy at the initiation of Atez/Bev. Regarding the Child‐Pugh classification, 205 (63.5%) patients were 5 points and 118 (36.5%) were 6 points, respectively. The median ALBI score was calculated to be −2.47 (−2.74 to −2.16). Accordingly, 130 (40.2%) patients were graded as mALBI 1, 82 (25.4%) as mALBI 2a, and 111 (34.4%) as mALBI 2b. Atez/Bev was used in the first‐line treatment for 182 (56.3%) patients. Nearly half of the patients (*n* = 152, 47.1%) were staged as B according to the BCLC stage criteria. Among the BCLC stage B patients, 127 (83.6%) patients were classified as beyond up‐to‐seven criteria.

**TABLE 1:** Patient characteristics before propensity score matching

Variables	Overall patients (*n* = 323)	Patients with viral infection (*n* = 165)	Patients with non‐viral infection (*n* = 158)	*p*‐value
Age, years	73.0 [68.0, 79.0]	72.0 [64.0, 78.0]	74.5 [70.0, 80.0]	0.005
Male, *n* (%)	259 (80.2)	128 (77.6)	131 (82.9)	0.27
PS, *n* (%)				
0	270 (83.6)	139 (84.2)	131 (82.9)	0.77
1	53 (16.4)	26 (15.8)	27 (17.1)	
Body mass index, (kg/m^2^)	23.1 [21.1, 25.7]	22.6 [20.7, 25.1]	23.6 [21.4, 26.1]	0.01
Etiology of liver diseases, *n* (%)				
HCV	103 (31.9)	103 (62.4)	0 (0.0)	<0.001
HBV	62 (19.2)	62 (37.6)	0 (0.0)	
Alcohol	59 (18.3)	0 (0.0)	59 (37.3)	
NAFLD	56 (17.3)	0 (0.0)	56 (35.4)	
Others	43 (13.3)	0 (0.0)	43 (27.2)	
Achieving SVR, *n* (%)^a^	77 (76.6)	77 (76.6)	0 (0.0)	NA
Antiviral therapy for HBV, *n* (%)^b^	40 (75.5)	40 (75.5)	0 (0.0)	NA
Platelet count (10^9^/L)	14.0 [10.8, 19.3]	13.4 [10.6, 17.7]	15.3 [11.3, 20.8]	0.01
Total bilirubin (mg/dl)	0.70 [0.60, 1.00]	0.80 [0.60, 1.00]	0.70 [0.58, 0.99]	0.12
Serum albumin (g/dl)	3.8 [3.4, 4.1]	3.9 [3.4, 4.2]	3.7 [3.4, 4.0]	0.02
Prothrombin time (%)	91.0 [83.0, 100.0]	91.0 [84.0, 100.0]	91.1 [82.0, 99.1]	0.22
Child‐Pugh score, *n* (%)				
5	205 (63.5)	109 (66.1)	96 (60.8)	0.36
6	118 (36.5)	56 (33.9)	62 (39.2)	
ALBI score	−2.47 [−2.74, −2.16]	−2.56 [−2.77, −2.16]	−2.36 [−2.67, −2.17]	0.05
mALBI grade, *n* (%)				
1	130 (40.2)	81 (49.1)	49 (31.0)	0.001
2a	82 (25.4)	30 (18.2)	52 (32.9)	
2b	111 (34.4)	54 (32.7)	57 (36.1)	
Treatment settings, *n* (%)				
First line	182 (56.3)	96 (58.2)	86 (54.4)	0.50
Later line	141 (43.7)	69 (41.8)	72 (45.6)	
BCLC stage, *n* (%)				
Intermediate stage	152 (47.1)	70 (42.4)	82 (51.9)	0.10
Advanced stage	171 (52.9)	95 (57.6)	76 (48.1)	
Beyond up‐to‐seven criteria	127 (83.6)	57 (81.4)	70 (85.4)	0.52
Extrahepatic spread, *n* (%)	110 (34.1)	60 (36.4)	50 (31.6)	0.41
Macrovascular invasion, *n* (%)	65 (20.1)	35 (21.2)	30 (19.0)	0.68
AFP≥400 ng/ml, *n* (%)	91 (28.2)	48 (29.1)	43 (27.2)	0.71
DCP≥400 mAU/ml^c^, n (%)	152 (47.5)	71 (43.3)	81 (51.9)	0.15

*Note*: Data are described as the median [interquartile range] or number (%). Data were missing for ^a^5, ^b^9, and ^c^3 patients, respectively.

Abbreviations: AFP, α‐fetoprotein; ALBI score, albumin‐bilirubin score; BCLC, Barcelona clinical liver cancer; DCP, des‐gamma‐carboxy prothrombin; HBV, hepatitis B virus; HCV, hepatitis C virus; mALBI grade, modified albumin‐bilirubin grade; NA, not applicable; NAFLD, non‐alcoholic fatty liver disease; PS, performance status; SVR, sustained virological response.

The patients with viral infection were younger, had a lower body mass index (BMI), a lower platelet count, higher level of albumin, better ALBI score, and better mALBI grade than those with non‐viral infection. Moreover, viral‐related HCC patients tended to more often have BCLC intermediate stage than non‐viral‐related HCC (*p* = 0.095).

Propensity score matching produced 126 matched pairs for patients with viral versus non‐viral infection. After matching, the significant differences in baseline demographic features did not exist between the two groups except for in the BMI and platelet count (Table S1).

### 3.2 The efficacy and safety outcome after propensity matching

The radiological response by RECIST v. 1.1 was complete response, partial response, stable disease, progressive disease, and not evaluable in 4 (3.2%), 22 (17.5%), 60 (47.6%), 21 (16.7%), and 19 (15.1%) patients with viral infection and in 3 (2.4%), 28 (22.2%), 56 (44.4%), 24 (19.0%), and 15 (11.9%) patients with non‐viral infection, respectively. Accordingly, the objective response rate was 20.6% and 24.6% in patients with viral and non‐viral infection, respectively, without a significant difference (*p* = 0.55). The disease control rate was also not significantly different between patients with viral and non‐viral infection (68.3% vs 69.0%, *p* = 1.00) (Table 2).

**TABLE 2:** A comparison of viral‐, and non‐viral‐related HCC patients after propensity score matching

Variables	Viral‐related HCC patients (*n* = 126)	Non‐viral‐related HCC patients (*n* = 126)	*p*‐value
CR	4 (3.2)	3 (2.4)	0.80
PR	22 (17.5)	28 (22.2)	
SD	60 (47.6)	56 (44.4)	
PD	21 (16.7)	24 (19.0)	
NE	19 (15.1)	15 (11.9)	
Objective response rate (%)	26 (20.6)	31 (24.6)	0.55
Disease control rate (%)	86 (68.3)	87 (69.0)	1.00

*Note*: Data were described as number (%) or median (95% CI) or percentage (95% CI). Tumor response was evaluated by RECIST ver.1.1.

Abbreviations: CI, confidence interval; CR, complete response; NE, non‐evaluable, PD, progressive disease; SD, stable disease; PR, partial response.

**FIGURE 2:**
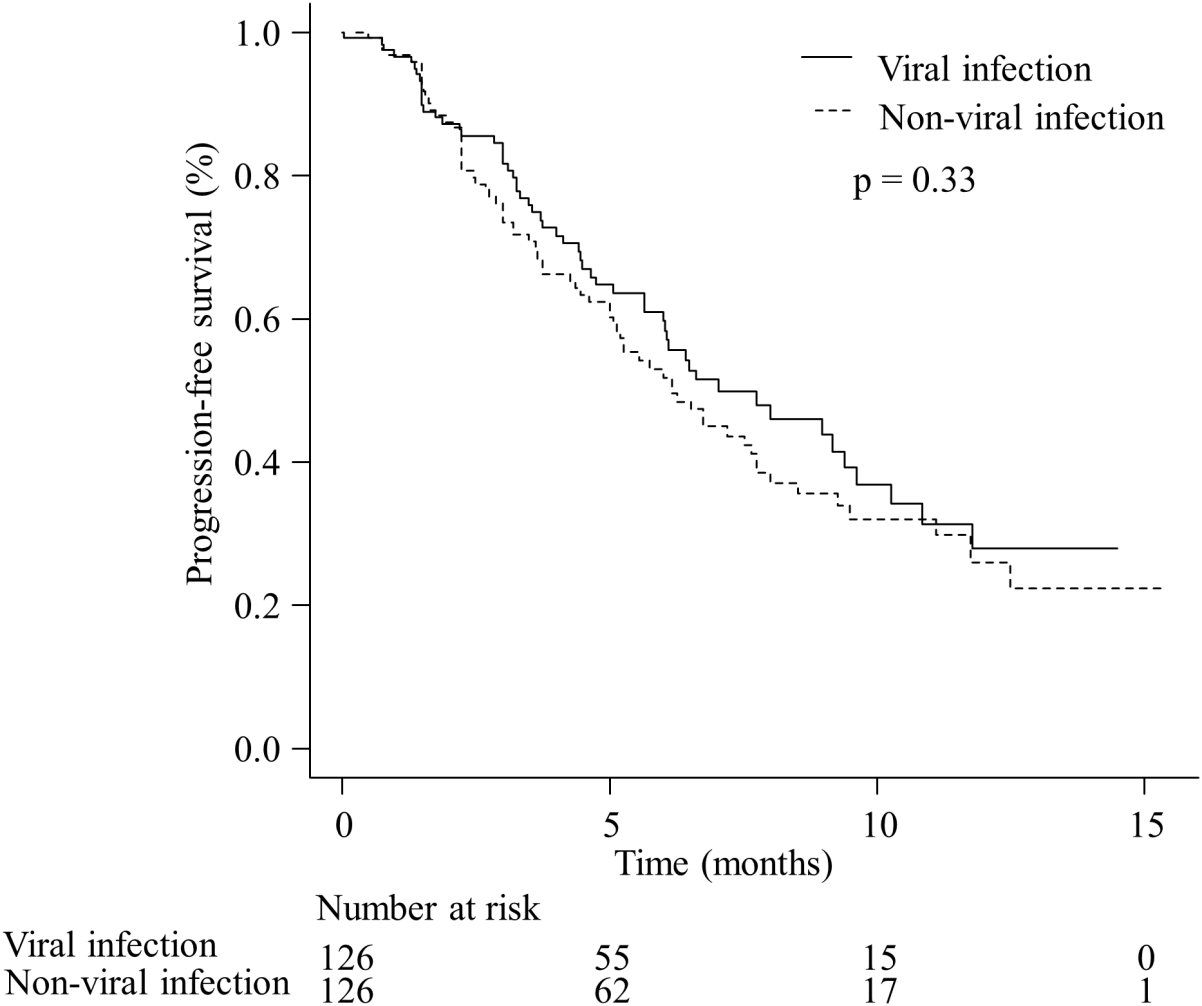
The progression‐free survival (PFS) of patients with viral and non‐viral infection. The median PFS was 7.0 (95% confidence interval [CI] 6.0–9.6) months and 6.2 (95% CI 5.1–7.8) months in viral and non‐viral patients, respectively, with a hazard ratio of 0.96 (95% CI 0.52–1.76). Significant differences did not note between the two groups (*p* = 0.33)

In the analysis of the PFS, 128 events (50.8%) were detected during the observation. The median PFS was 7.0 months (95% CI 6.0–9.6) in patients with viral infection whereas it was 6.2 months (95% CI 5.1–7.8) in patients with non‐viral infection, respectively, with an HR of 0.96 (95% CI 0.52–1.76). No significant difference between the two groups was not found (*p* = 0.33; Figure 2). After the end of Atez/Bev treatment, 38 (70.4%) and 56 (78.9%) patients with viral and non‐viral infection received any subsequent treatments. The proportion of any subsequent treatments were not significantly differed between them (*p* = 0.30).

**FIGURE 3:**
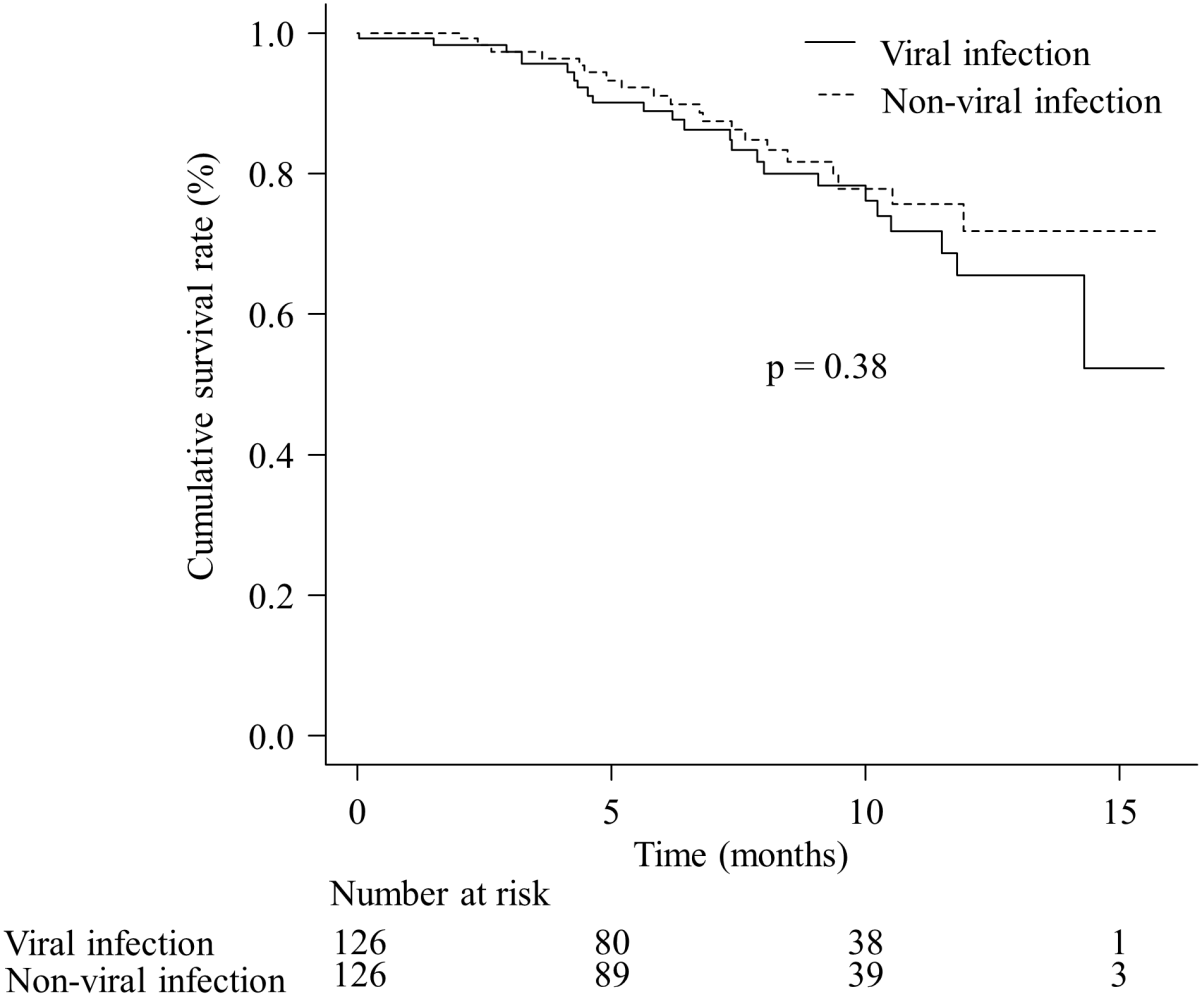
The survival curve of patients with viral and non‐viral infection. The median overall survival (OS) in patients with viral and non‐viral infection was not reached. The 3‐, 6‐, and 12‐month OS rates were 97.4% (95% confidence interval [CI] 92.2%–99.2%), 88.8% (95% CI 80.6%–93.7%), and 65.5% (95% CI 50.8%–76.8%) in patients with viral infection and 97.3% (95% CI 92.0%–99.1%), 91.1% (95% CI 83.5%–95.3%), and 71.7% (95% CI 57.3%–81.9%) in patients with non‐viral infection, respectively. No significant differences were found between the two groups (*p* = 0.38, hazard ratio 0.76, 95% CI 0.42–1.39)

In the analysis of the OS, 44 deaths (17.5%) were found during the observation. The median OS was not reached in both the groups. The 3‐, 6‐, and 12‐month OS rates were 97.4% (95% CI 92.2%–99.2%), 88.8% (95% CI 80.6%–93.7%), and 65.5% (95% CI 50.8%–76.8%) in patients with viral infection and 97.3% (95% CI 92.0%–99.1%), 91.1% (95% CI 83.5%–95.3%), and 71.7% (95% CI 57.3%–81.9%) in patients with non‐viral infection, respectively. No significant differences were observed between the two groups (*p* = 0.38; HR 0.76, 95% CI 0.42–1.39; Figure 3).

Next, we investigate the PFS and OS in the patients treated with Atez/Bev as the first‐line treatment and later‐line treatment, finding that the significant extents were not differed between viral‐, and non‐viral‐related HCC patients. The Kaplan–Meier curves are shown in Figures S1 and S2a,b. Furthermore, we analyzed the PFS and OS according to each etiology of underlying liver disease, noting no significant differences by etiology (HCV, HBV, alcohol, NAFLD and others). The PFS and OS curves are shown in Figure S2. The results of comparison of NAFLD and other etiologies (HCV, HBV, alcohol, and others) were shown in Figure S4.

The most commonly experienced AEs in propensity‐score‐matched cohort were protein urea, secondary to fatigue, appetite loss, hypertension, and liver injury during the observation period. We compared the incidence and severity of each AE in both two groups, resulting in no significant differences (Table 3).

**TABLE 3:** Incidence and degree of adverse events in viral‐, and non‐viral‐related HCC patients after propensity score matching

Variables		Viral‐related HCC patients (*n* = 126)	Non‐viral‐related HCC patients (*n* = 126)	*p*‐value
Diarrhea	Any	8 (6.3)	9 (7.1)	1.00
	Grade ≥3	3 (2.4)	0 (0.0)	0.25
Liver injury	Any	15 (11.9)	16 (12.7)	1.00
	Grade ≥3	4 (3.2)	5 (4.0)	1.00
Hypertension	Any	20 (15.9)	18 (14.3)	0.86
	Grade ≥3	8 (6.3)	3 (2.4)	0.22
Appetite loss	Any	23 (18.3)	29 (23.0)	0.44
	Grade ≥3	3 (2.4)	5 (4.0)	0.72
Protein urea	Any	30 (23.8)	26 (20.6)	0.65
	Grade ≥3	16 (12.7)	10 (7.9)	0.30
Fever	Any	9 (7.1)	10 (7.9)	1.00
	Grade ≥3	3 (2.4)	1 (0.8)	0.62
Fatigue	Any	26 (20.6)	29 (23.0)	0.76
	Grade ≥3	2 (1.6)	2 (1.6)	1.00
Gastrointestinal tract bleeding	Any	4 (3.2)	2 (1.6)	0.68
	Grade ≥3	3 (2.4)	1 (0.8)	0.62
Ascites, hepatic edema	Any	7 (5.6)	15 (11.9)	0.18
	Grade ≥3	1 (0.8)	5 (4.0)	0.21

## 4 DISCUSSION

We compared the baseline characteristics between the patients with viral and non‐viral infection, revealing that patients with viral infection were significantly younger with a better liver function than those with non‐viral infection. In addition, the viral‐related‐HCC patients tended to have a higher percentage of BCLC advanced stage than those with non‐viral infection. We conducted propensity score matching to correct for these imbalances in baseline characteristics, demonstrating that significant differences in pretreatment factors associated with efficacy and safety did not note between the two groups. In addition, we also investigated the PFS and OS in patients receiving Atez/Bev as the first‐line treatment and later treatment, indicating that there were not significant differences between viral‐, and non‐viral HCC patients. We also compared the PFS and OS according to each etiology of liver disease, showing that the significant extent was not found.

The present etiology‐based study showed that Atez/Bev had good efficacy and safety for HCC patient with non‐viral infection as well as those with viral infection. This indicates that underlying liver diseases did not affect the clinical outcome of Atez/Bev treatment. A real‐world data indicated that underlying liver diseases did not affect the clinical outcome of sorafenib.^12^ As for lenvatinib, whether or not liver etiology influenced on the therapeutic outcome remains controversial. Two real‐world studies reported that liver etiology did not associate with overall survival^13^, ^14^ while another two studies showed the overall survival is better in patients with non‐viral infection than in those with viral infection.^15^, ^16^ However, no studies had compared Atez/Bev in patients with viral and no‐viral infection in real‐world settings. As far as we know, this is the first study investigating the association between the clinical outcome of Atez/Bev and the etiology of liver disease based on real‐world data.

Pfister et al.^5^ carried out a meta‐analysis of three RCT trials (CheckMate‐459,^17^ Imbrave150,^1^ and KEYNOTE‐240^18^), indicating that patients with viral infection benefitted from ICI treatment compared to standard‐care treatment, while those with non‐viral infection did not benefit from this treatment. Haber et al.^6^ performed a systematic review of phase‐three RCTs (reported from 2002 to 2020) and examined the association between the liver etiology and outcome of ICI treatment and between the liver etiology and outcome of tyrosine kinase inhibitor (TKI)/anti‐VEGF therapies. The efficacy of ICIs between viral‐ and non‐viral‐related HCC significantly differ between viral‐ and non‐viral‐related HCC, while the liver etiology was not associated with the outcome of TKI/anti‐VEGF therapies.^6^

The present results seem to be in disagreement with these previous studies and the results of the subgroup analysis of Imbrave150.^1^, ^4^ While we cannot concretely explain this difference, some possible reasons may be hypothesized. First, the treatment method (ICI monotherapy/ICI plus VEGF inhibitor combination therapy) and treatment settings (first‐, and second‐line treatment) differed among the three RCT trials.^1^, ^17^, ^18^ Second, ICI treatment was compared to sorafenib or best supportive care (BSC) in three RCT trials,^1^, ^17^, ^18^ whereas we compared the therapeutic outcome of Atez/Bev in patients with viral and non‐viral infection. This means that the control group differed between the three RCT trials (sorafenib or BSC) and the present study (non‐viral patients). Third, more than 70% patients with viral infection received antiviral therapy in the current study. The persistent liver inflammation after HCV eradication was mainly associated with hepatic steatosis,^19^, ^20^ which were a risk factor of carcinogenesis.^21^ Hence, antiviral therapy may affect the present results. Fourth, while the updated subpopulation analysis of Imbrave150^4^ showed that the OS in non‐viral patients receiving Atez/Bev was comparable to the value in those receiving sorafenib (HR 1.05, 95% CI 0.68–1.63), details concerning baseline characteristics were not clarified. Among non‐viral‐related HCC patients, the statistical comparison of baseline characteristics between Atez/Bev and sorafenib groups will be required to assess the therapeutic outcome. A further study with a greater focus on the baseline characteristics stratified by the liver disease etiology is therefore suggested.

Pfister et al^5^ reported that NASH‐driven HCC was not benefitted from immunotherapy targeted at PD‐1 due to impaired immune surveillance, which were presumed to disagree with the present results. This difference is possibly due to the presence of combined anti‐VEGF therapy. VEGF inhibited dendric cell maturation, reduced T‐cell tumor infiltration, and increase immune‐suppressive cells such as myeloid derived suppressor cells and regulatory T cells in the tumor microenvironment, resulting in immune‐suppressive effects.^22^ In addition, bevacizumab not only enhanced the efficacy of immunotherapies but also showed the antitumor effect for HCC.^23^ A further study will be required to investigate whether or not NASH‐driven HCC respond to combination of immunotherapy and anti‐VEGF therapy.

Recently, the HIMALAYA phase 3 study, which examined the efficacy and safety of tremelimumab and durvalumab in comparison to sorafenib, reported that tremelimumab and durvalumab achieved a good survival in HBV patients (HR 0.64, 95% CI 0.48–0.86), and non‐viral patients (HR 0.74, 95% CI 0.57–0.95) but not in HCV patients (HR 1.06, 95% CI 0.76–1.49).^24^ According to the PFS and preliminary interim OS results of COSMIC‐312 trial^25^ evaluating the therapeutic efficacy of cabozantinib and atezolizumab versus sorafenib in first‐line treatment, cabozantinib and atezolizumab improved OS in HBV patients (HR 0.53, 95% CI 0.33–0.87) but did not in HCV patients (HR 1.10, 95% CI 0.72–1.68) or non‐viral patients (HR 1.18, 95% CI 0.78–1.79). Given these results, further analyses will be needed to investigate whether or not ICI treatment was unlikely to induce a response in non‐viral‐related HCC patients.

After matching, the platelet count and BMI were lower in viral patients than in non‐viral patients, probably due to disease‐specific differences. In general, the platelet count was lower in HBV or HCV patients than that in nonB‐, nonC‐ patients. Indeed, the cut‐off value of HCV‐related cirrhosis^26^ was lower than that of NAFLD‐related cirrhosis.^27^ Regarding the BMI, this difference was because the carcinogenesis of non‐viral‐related HCC was strongly associated with metabolic disease, including obesity, hypertension, and diabetes mellitus, resulting in a higher BMI in non‐viral HCC patients than in viral HCC patients. Furthermore, while the BCLC stage,^28^ mALBI grade,^28^ early AFP response,^29^, ^30^ neutrophil‐to‐lymphocyte ratio,^31^, ^32^ and early bev interruption^33^ were reported to be associated with the outcome of Atez/Bev, whether or not the platelet count and BMI influenced the outcome remains uncertain. Accordingly, we believe that it is not appropriate to correct the imbalance in the platelet count and BMI between the two groups.

In the current study, about 70% patients received any subsequent therapies after the end of Atez/Bev treatment. Given that post‐progression survival was strongly correlated with OS,^34^ subsequent therapies play an important role in prolongation of survival. While the proportion of receiving the subsequent therapies after sorafenib^35^ and lenvatinib^36^ are reported to be about 50%, the previous real‐world studies^37^, ^38^ showed that about 70% patients received it after Atez/Bev treatment, which was consistent to the present study.

While there was no RCT comparing the efficacy and safety of Atez/Bev and lenvatinib in patients with unresectable HCC patients, comparative studies based on real‐world data have recently been reported. Kim et al reported that the OS and PFS was not significantly differed between the Atez/Bev and lenvatinib while the objective response was better in lenvatinib‐treated patients than Atez/Bev‐treated patients with non‐viral infection.^39^ Two studies reported by Hiraoka et al^40^ and Maesaka et al^41^ showed that there were no differences in the OS and PFS between patients treated with Atez/Bev and lenvatinib. Because the number of the participated patients was small and the observation period was short, further comparative studies with sufficient cases and follow‐up period were warranted.

There are some important limitations in the current study. First, this study was analyzed in a retrospective manner. Second, although we conducted propensity score matching, some unintentional bias may still have been included. Third, there is the relatively small number of objects with a short observation period. For example, although the statistical significance was not observed, the survival curve might be distinct in patients receiving Atez/Bev as the first‐line treatment (Figure S1). Including more patients with a long‐term follow‐up might influence the present findings. Fourth, there were about 70% patients who received antiviral therapy in the current study. Because steatohepatitis is associated with the carcinogenesis after the eradication of HCV, as we mention above, antiviral therapy might affect the present results. Another limitation is the high percentage of patients receiving subsequent therapies after the Atez/Bev treatment. About 50% patients have BCLC intermediate stage in the present cohort while only 10% patients have BCLC intermediate stage in Imbrave150. Given that BCLC intermediate stage HCC patients tend to receive the post‐progression treatment comparing to BCLC advanced stage HCC patients, the subsequent treatment might affect the present OS results.

In conclusion, the present etiology‐based study revealed that Atez/Bev showed good efficacy and safety for HCC patient with non‐viral infection as well as those with viral infection.

## AUTHOR CONTRIBUTIONS

**Takeshi Hatanaka:** Conceptualization (equal); data curation (equal); formal analysis (equal); investigation (equal); writing – original draft (equal). Satoru Kakizaki**:** Conceptualization (equal); data curation (equal); investigation (equal); writing – review and editing (equal). **Atsushi Hiraoka:** Conceptualization (equal); data curation (equal); writing – review and editing (equal). **Toshifumi Tada:** Conceptualization (equal); data curation (equal); writing – review and editing (equal). **Masashi Hirooka:** Data curation (equal). **Kazuya Kariyama:** Data curation (equal). **Joji Tani:** Data curation (equal). **Masanori Atsukawa:** Data curation (equal). **Koichi Takaguchi:** Data curation (equal). **Ei Itobayashi:** Data curation (equal). **Shinya Fukunishi:** Data curation (equal). **Kunihiko Tsuji:** Data curation (equal). **Toru Ishikawa:** Data curation (equal). **Kazuto Tajiri:** Data curation (equal). **Hironori Ochi:** Data curation (equal). **Satoshi Yasuda:** Data curation (equal). **Hidenori Toyoda:** Data curation (equal). **Chikara Ogawa:** Data curation (equal). **Takashi Nishimura:** Data curation (equal). **Noritomo Shimada:** Data curation (equal). **Kazuhito Kawata:** Data curation (equal). **Hisashi Kosaka:** Data curation (equal). **Takaaki Tanaka:** Data curation (equal). **Hideko Ohama:** Data curation (equal). **Kazuhiro Nouso:** Data curation (equal). **Asahiro Morishita:** Data curation (equal). **Akemi Tsutsui:** Data curation (equal). **Takuya Nagano:** Data curation (equal). **Norio Itokawa:** Data curation (equal). **Tomomi Okubo:** Data curation (equal). **Taeang Arai:** Data curation (equal). **Michitaka Imai:** Data curation (equal). **Atsushi Naganuma:** Conceptualization (equal); data curation (equal); investigation (equal); writing – review and editing (equal). **Yohei Koizumi:** Data curation (equal). **Shinichiro Nakamura:** Data curation (equal). **Kouji Joko:** Data curation (equal). **Masaki Kaibori:** Data curation (equal). **Hiroko Iijima:** Data curation (equal). **Yoichi Hiasa:** Data curation (equal). **Takashi Kumada:** Conceptualization (equal); investigation (equal); writing – review and editing (equal).

## CONFLICT OF INTEREST

Takeshi Hatanaka has received honoraria from Eisai. Atsushi Hiraoka has received honoraria from Eli Lilly, Bayer, and Chugai. Toshifumi Tada has received honoraria from AbbVie, and Eisai. Hiroko Iijima has received research grants from Abbvie, Otsuka, and Sumitomo Dainippon Pharma. Takashi Kumada has received honoraria from Eisai. None of the other authors have potential conflicts of interest to declare.

## ETHICS STATEMENT

All research procedures approved by the Institutional Ethics Committee of Ehime Prefectural Central Hospital (IRB No. 30‐66) (UMIN000043219) and were undertaken in accordance with the Declaration of Helsinki. All patients agreed and gave written consent for anonymous use of their clinical data for scientific research.

## Supporting information


Appendix S1

Table S1.

Figure S1.

Figure S2.

Figure S3.

Figure S4.

## Data Availability

The datasets in this study are available from the corresponding author upon reasonable request.
