# Supplementary material for: Comparative efficacy and safety of atezolizumab and bevacizumab between hepatocellular carcinoma patients with viral and non‐viral infection: A Japanese multicenter observational study
Source: Cancer Med. 2022 Oct 13;12(5):5293–303. doi: 10.1002/cam4.5337 (PMC10028018; doi:10.1002/cam4.5337)
Supplement: Supplementary file 1 — Appendix S1 [file CAM4-12-5293-s001.docx]

**Supplemental Figure Legends**

**Supplemental Figure 1.** (a) The progression-free survival (PFS) of patients receiving atezolizumab combined with bevacizumab (Atez/Bev) as the first-line treatment. The median PFS was 8.9 (95% confidence interval [CI] 5.6-not applicable) months and 6.2 (95% CI 5.0-8.0) months in patients with viral and non-viral infection, respectively. There were no significant differences between the two groups (p=0.22, hazard ratio [HR] 1.38, 95% CI 0.82-2.30). (b) The survival curve of patients receiving Atez/Bev as the first-line treatment. The 3-, 6-, and 12-month overall survival rates were 96.8% (95% CI 87.8%-99.2%), 88.4% (95% CI 75.6%-94.7%), and 58.4% (95% CI 29.4%-78.9%) in patients with viral infection and 96.7% (95% CI 87.6%-99.2%), 88.9% (95% CI 76.8%-94.9%), and 74.7% (95% CI 55.3%-86.6%) in patients with non-viral infection, respectively. No significant differences were found between the two groups (p=0.46, HR 0.72, 95% CI 0.31-1.71).

**Supplemental Figure 2.** (a) The progression-free survival (PFS) of patients receiving atezolizumab combined with bevacizumab (Atez/Bev) as the later-line treatment. The median PFS was 6.4 (95% confidence interval [CI] 4.4-not applicable) months and 6.3 (95% CI 3.8-8.5) months in patients with viral and non-viral infection, respectively. There were no significant differences between the two groups (p=0.78, hazard ratio [HR] 1.07, 95% CI 0.66-1.73). (b) The survival curve of patients receiving Atez/Bev as the later-line treatment. The 3-, 6-, and 12-month overall survival rates were 98.1% (95% CI 87.1%-99.3%), 89.6% (95% CI 76.8%-95.6%), and 68.3% (95% CI 49.5%-81.3%) in patients with viral infection and 98.0% (95% CI 86.9%-99.7%), 93.7% (95% CI 81.6%-97.9%), and 70.3% (95% CI 49.6%-83.7%) in patients with non-viral infection, respectively. No significant extents were noted between the two groups (p=0.67, HR 0.83, 95% CI 0.37-1.90).

**Supplemental Figure 3.** (a) The progression-free survival (PFS) of patients according to each etiology of liver disease. The median PFS was 7.0 (95% confidence interval [CI] 5.1-10.3), 8.0 (95% CI 4.6-10.9), 6.8 (95% CI 5.3-9.5), 6.3 (95% CI 5.0-11.8), 6.3 (95% CI 5.0-11.8), and 4.5 (95% CI 3.0-7.5) months in hepatitis C virus (HCV)-, hepatitis B virus (HBV)-, alcohol-, non-alcoholic fatty liver disease (NAFLD)- and other etiology-related hepatocellular carcinoma (HCC) patients, respectively. No significant differences were observed between each etiology of liver disease (p=0.64). (b) The survival curve of patients based on each etiology of liver disease. The 12-month overall survival rates were 66.7% (95% CI 46.9%-80.6%), 64.6% (95% CI 42.0%-80.2%), 75.2% (95% CI 53.9%-87.6%), 63.8% (95% CI 35.1%-82.5%), and 77.5% (95% CI 50.1%-91.0%) in HCV-, HBV-, alcohol-, NAFLD-, and other etiology-related hepatocellular carcinoma (HCC) patients, respectively. There were no significant differences among the etiologies of liver disease (p=0.92).

**Supplemental Figure 4.** (a) The progression-free survival (PFS) of patients with non-alcoholic fatty liver disease (NAFLD) and other etiologies (hepatitis C virus, hepatitis B virus, alcohol, and others). The median PFS was 6.3 (95% CI 5.0-11.8) and 6.6 (95% CI 5.6-8.5) months in patients with NAFLD and other etiologies. There was not significant difference between the two groups (p=0.86, hazard ratio [HR] 0.96, 95% CI 0.62-1.49). (b) The survival curve of patients with NAFLD and other etiologies. The 12-month overall survival rates were 63.8% (95% CI 35.1%-82.5%), and 69.8% (95% CI 59.2%-78.2%) in patients with NAFLD and other etiologies. The significant extents were not differed between them (p=0.97, HR 1.01, 95% CI 0.47-2.18).
