# Supplementary figures and images for: Comparative efficacy and safety of atezolizumab and bevacizumab between hepatocellular carcinoma patients with viral and non‐viral infection: A Japanese multicenter observational study
Source: Cancer Med. 2022 Oct 13;12(5):5293–303. doi: 10.1002/cam4.5337 (PMC10028018; doi:10.1002/cam4.5337)

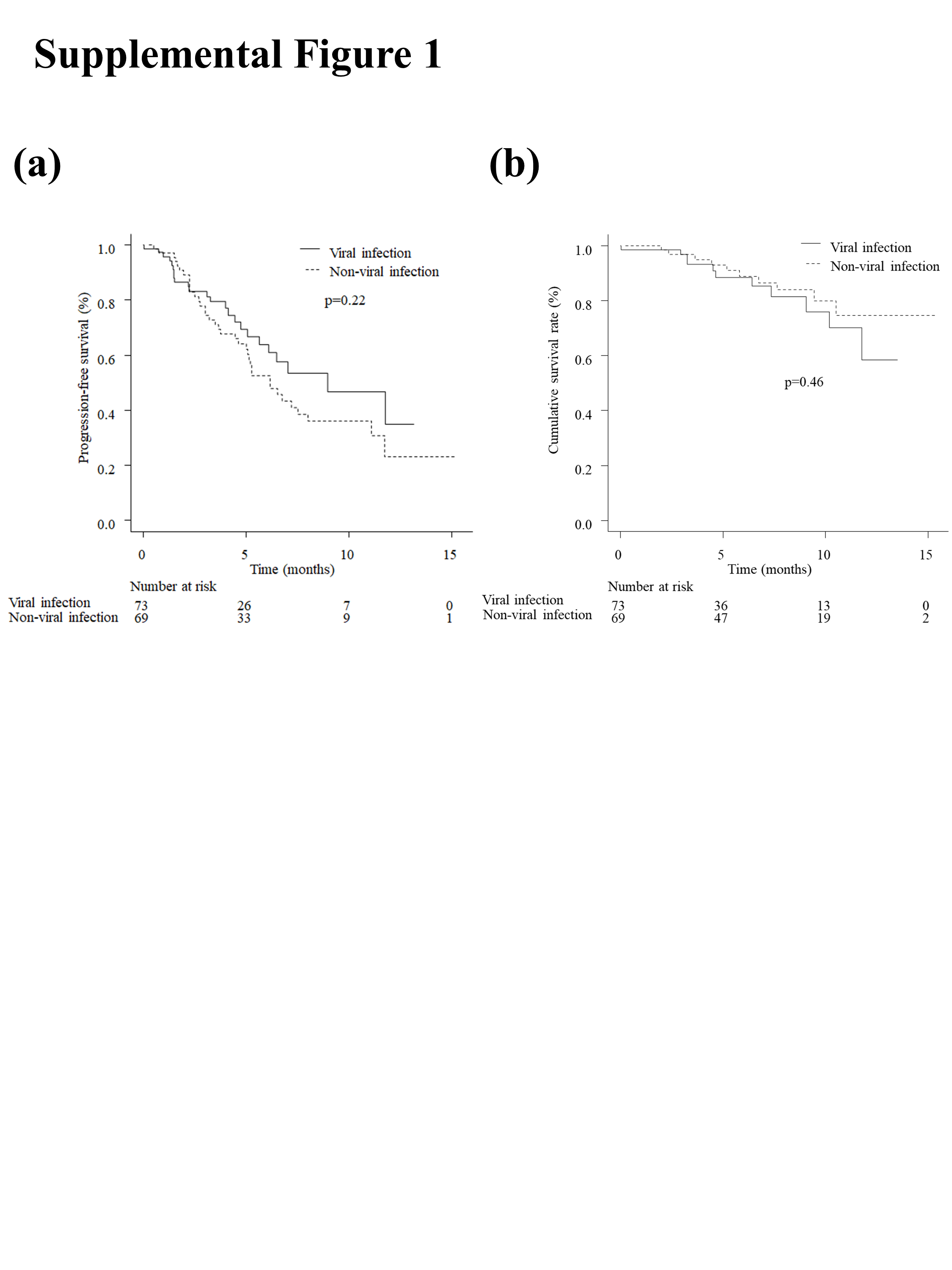

Supplement: Supplementary file 3 — Figure S1. [file CAM4-12-5293-s003.TIF]

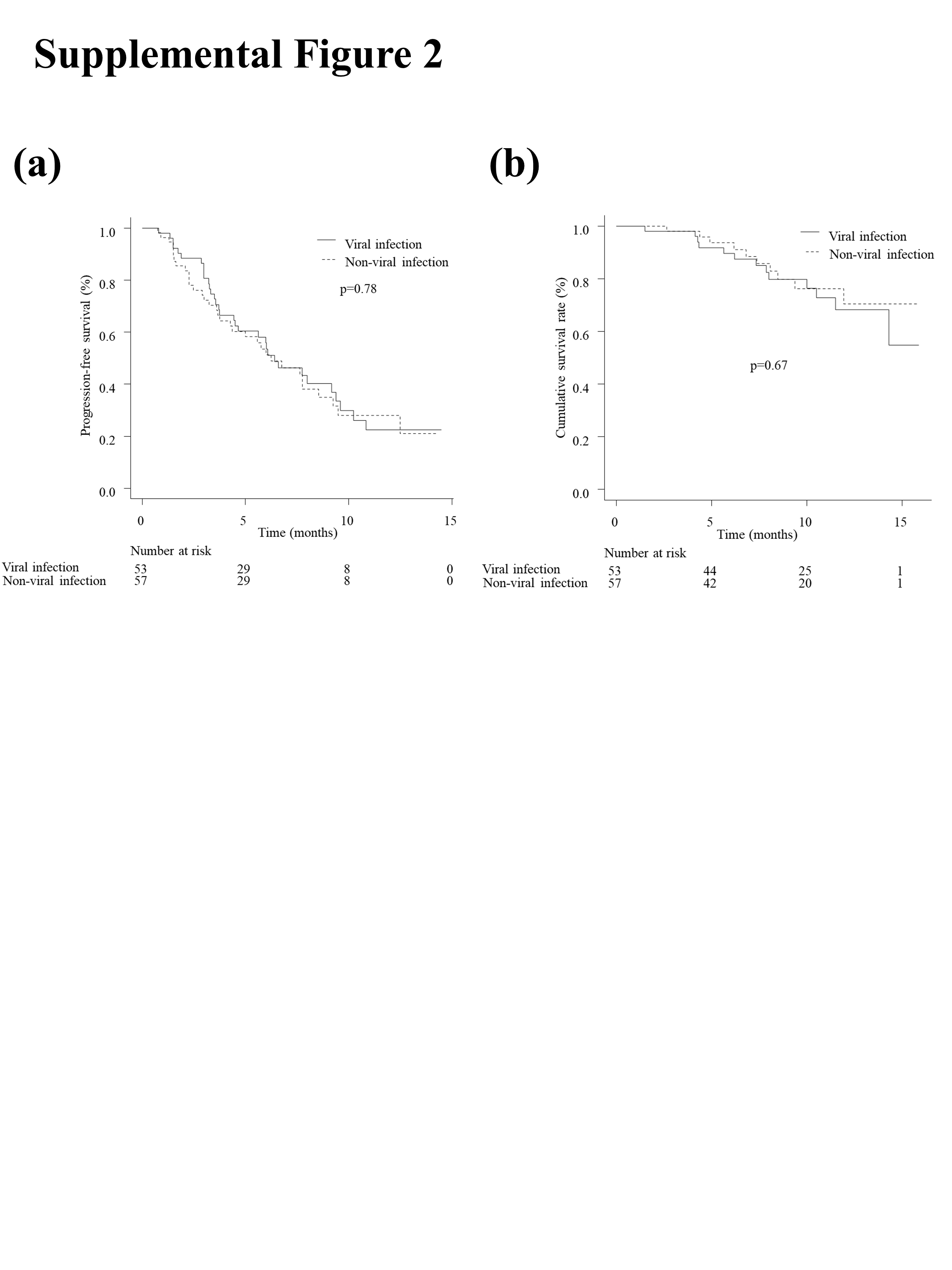

Supplement: Supplementary file 4 — Figure S2. [file CAM4-12-5293-s005.TIF]

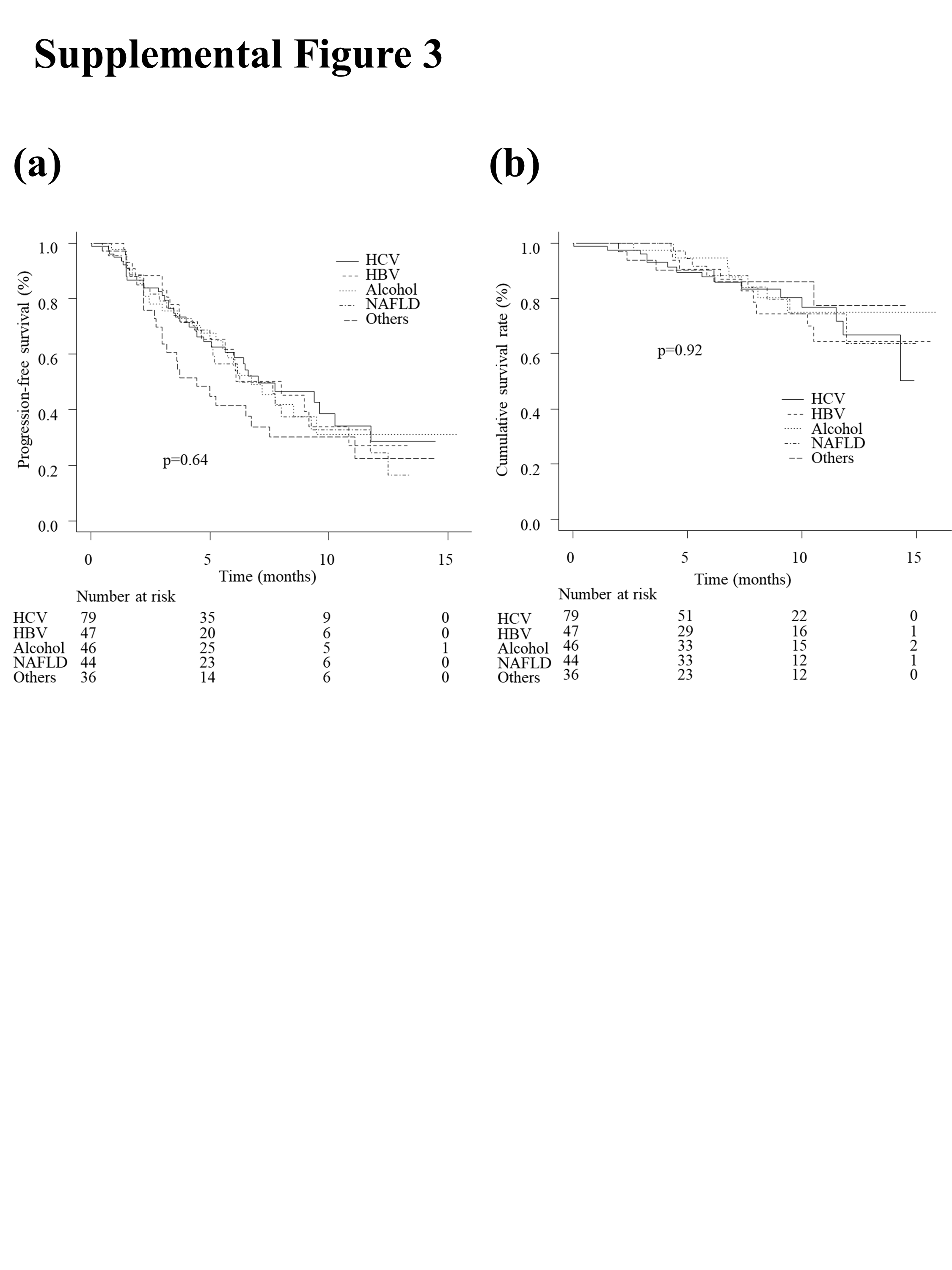

Supplement: Supplementary file 5 — Figure S3. [file CAM4-12-5293-s002.TIF]

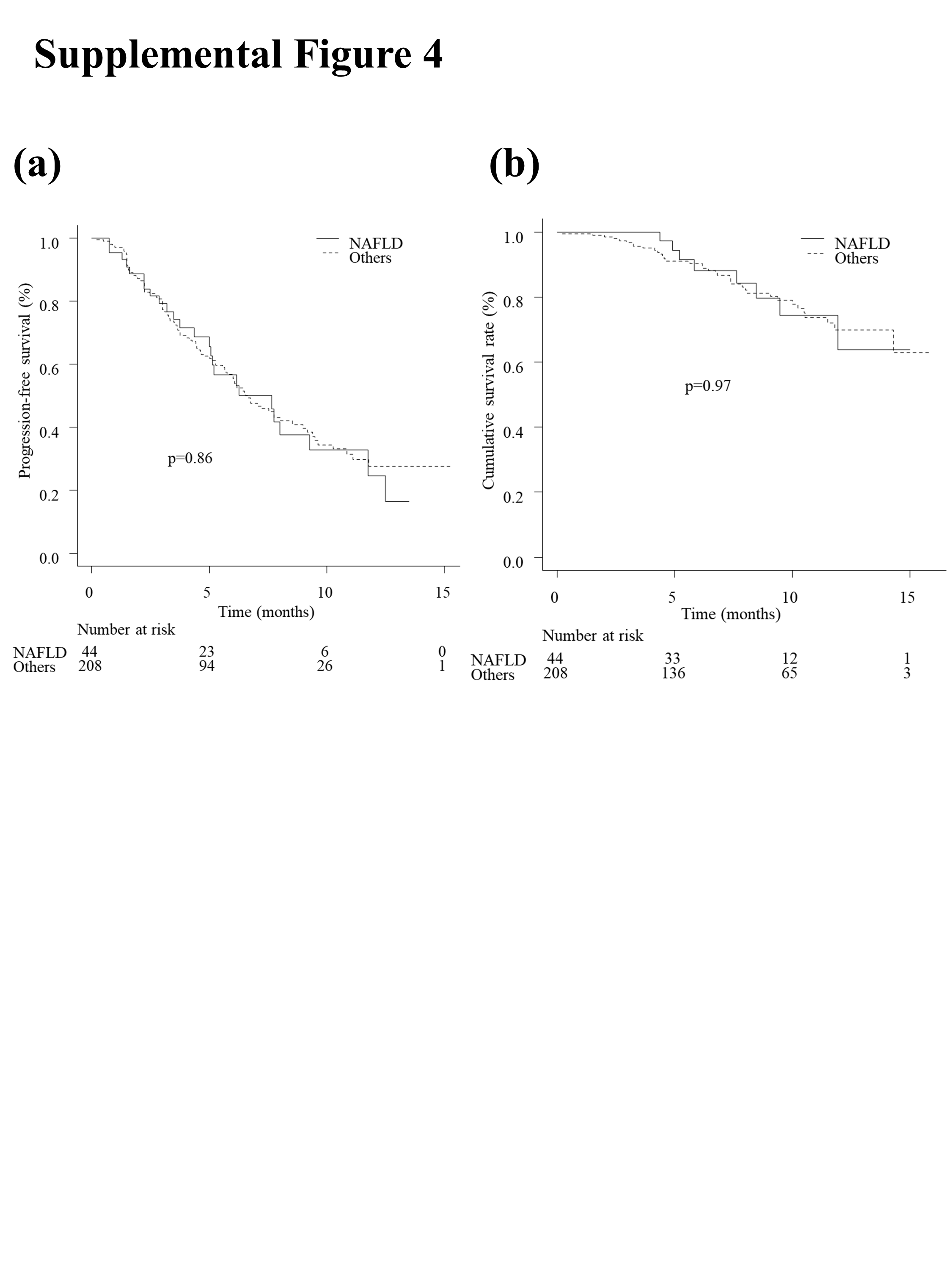

Supplement: Supplementary file 6 — Figure S4. [file CAM4-12-5293-s006.TIF]
